# Thy1 retroviral reporters: a versatile platform for congenic background-independent adoptive transfer and CD8^+^ T cell differentiation studies

**DOI:** 10.3389/fimmu.2026.1917932

**Published:** 2026-09-16

**Authors:** Chunlong Liu, Chunling Liu, Jing Zhong, Xiaodi Yang, Dapeng Wang

**Affiliations:** 1 Department of Pathophysiology, School of Basic Medicine, Bengbu Medical University, Bengbu, China; 2 Scientific Research Center, Bengbu Medical University, Bengbu, China; 3 Department of Microbiology and Parasitology, School of Basic Medicine, Bengbu Medical University, Bengbu, China; 4 Anhui Key Laboratory of Infection and Immunity, Bengbu Medical University, Bengbu, China; 5 Key Laboratory of Cardiovascular and Cerebrovascular Diseases, Bengbu Medical University, Bengbu, China

**Keywords:** adoptive transfer, CD8+ T cell differentiation, retroviral vector, T-bet, thy1

## Abstract

Adoptive T cell transfer remains a fundamental technique for dissecting the molecular mechanisms governing T cell differentiation. A successful adoptive-transfer experiment typically relies on labor-intensive construction of specific congenic mouse strains to distinguish donor from host cells. This limitation may be addressed by a readily detectable surface reporter. Thy1 is a candidate, but it remains unclear whether it is a reliable reporter for monitoring adoptively transferred CD8^+^ T cell differentiation because Thy1 is a functional protein involved in thymic development and cell activation. To address this question, we systematically evaluated the reliability, stability, and functional neutrality of Thy1.1 and Thy1.2, two Thy1 isoforms, as adoptive-transfer reporters during CD8^+^ T cell differentiation *in vivo*. Our results show that endogenous Thy1 expression remains stable across the differentiation stages examined. In the experimental settings tested, exogenous Thy1 reporter expression did not measurably alter the assessed differentiation phenotypes or donor-cell recovery. We further constructed and validated a Thy1 reporter-based platform that captured phenotypes associated with both loss-of-function (shRNA) and gain-of-function (cDNA) modulation of the transcription factor T-bet. This system provides a practical option for congenic background-independent adoptive transfer and genetic studies of CD8^+^ T cell differentiation.

## Introduction

Adoptive T cell transfer has long been an indispensable tool for investigating both CD4^+^ and CD8^+^ T cell biology, allowing for the precise tracking of antigen-specific cells within controlled physiological environments (1–3). This approach is particularly critical for CD8^+^ T cell research because, unlike CD4^+^ T cell differentiation—which is largely cytokine-dependent and amenable to *in vitro* modeling—CD8^+^ T cell differentiation is driven primarily by inflammatory signals and remains highly dependent on complex *in vivo* environmental cues.

To accomplish this goal, researchers frequently employ the adoptive transfer of TCR-transgenic T cells into host mice to observe their differentiation kinetics in a physiological setting (4). When coupled with viral-mediated delivery of nucleic acids—such as shRNA, gRNA, or cDNA—this approach allows for the precise interrogation of gene function via robust gain-of-function or loss-of-function methodologies (5).

A critical technical prerequisite for adoptive transfer is the ability to reliably distinguish donor T cells from the endogenous host population. This has historically been achieved using congenic markers such as the leukocyte common antigen CD45 (Ptprc) (6) or the GPI-anchored protein Thy1 (CD90) (7). However, the success of a standard adoptive transfer experiment is strictly dependent on the mismatch between host and donor congenic backgrounds (e.g., CD45.1+ donors into CD45.2+ hosts, or Thy1.1^+^ donors into Thy1.2 hosts). This requirement imposes significant logistical constraints, as experimental designs are often delayed or restricted by the need to backcross transgenic or knockout lines onto specific congenic backgrounds—a process that can take several generations (8).

To circumvent these challenges, a surface protein marker might serve as an attractive alternative. In this study, we focused on Thy1, which was sporadically used as a reporter in T cell-based tumor immunity studies (9–12). We systematically evaluated the reliability and feasibility of Thy1 reporters in the context of CD8^+^ T cell differentiation *in vivo*.

## Materials and methods

### Mice and virus

C57BL/6N mice were purchased from Vital River Laboratory Animal Technology Co., Ltd. P14 TCR-transgenic mice and the lymphocytic choriomeningitis virus (LCMV) Armstrong strain (LCMV-Arm) and clone 13 strain (LCMV-C13) were generously provided by Dr. Lilin Ye (Institute of Immunology, Third Military Medical University, Chongqing, China). To minimize genetic background differences, P14 mice were backcrossed to C57BL/6N mice for more than five generations before use. All mice were maintained under specific pathogen-free conditions, and all animal experiments were conducted in accordance with the institutional guidelines of Bengbu Medical University. LCMV-Arm was propagated in BHK-21 cells, and viral titers were determined by plaque assay using Vero cells.

### Thy1 reporter retroviral vectors

The retroviral vector MIGR1 (Addgene, 27490) was used as the backbone for Thy1 reporter-vector construction. For the Thy1.1-based RNAi vector, the human miR-30-based shRNAmir targeting mouse *Cd4* or *Tbx21* mRNA, the PGK promoter, and Thy1.1 cDNA replaced the endogenous IRES-GFP cassette. For the Thy1.2-based overexpression vector, GFP cDNA was replaced with Thy1.2 cDNA, and mouse *Tbx21* cDNA was cloned upstream of the IRES-Thy1.2 element. In the RNAi experiments, *Cd4*-targeting shRNA was used as a control because *Cd4* is not expressed in P14 CD8^+^ T cells.

### Retrovirus packaging

293T cells were cultured in complete DMEM supplemented with 10% fetal bovine serum. One day before transfection, cells were plated in 6-well plates or 10-cm dishes to reach 70-80% confluence at transfection. Thy1 reporter retroviral vectors and the packaging plasmid pCL-Eco (Addgene, 12371) were co-transfected for 6 h. The transfection medium was then replaced with fresh complete medium. Viral supernatants were collected every 24 h for three consecutive days.

### Retroviral transduction of CD8^+^ T cells

CD8^+^ T cells were activated with anti-CD3 (clone 145-2C11) and anti-CD28 (clone 37.51) for 20 h. After the medium was removed, retroviral supernatant was added and cells were spin-infected at 2,000g for 1 h. The virus-containing supernatant was removed 4 h later. Cells were washed and either adoptively transferred into naive mice for infection experiments or maintained *in vitro*.

### CD8^+^ T cell differentiation *in vivo*

Naive P14 T cells were isolated from P14 transgenic mice carrying either the Thy1.1^+/+^ or Thy1.2^+/+^ congenic allele. Cells were activated for 20 h on plates coated with 30 µg/ml goat anti-hamster IgG (H+L) secondary antibody (Thermo Fisher Scientific, catalog no. 31115) in the presence of 1 µg/ml anti-mouse CD3ϵ antibody (BioLegend, catalog no. 100340) and 1 µg/ml anti-mouse CD28 antibody (BioLegend, catalog no. 102116). Thy1.1^+/+^ or Thy1.2^+/+^ P14 cells were transduced with a Thy1.2 reporter retrovirus expressing *Tbx21* cDNA or mock control, or with a Thy1.1 reporter retrovirus carrying shRNA targeting *Tbx21* or *Cd4* (control). Transduced cells were transferred into naive C57BL/6 mice carrying the same endogenous Thy1 congenic allele as the P14 donor cells. Mice were infected intraperitoneally with 2 × 10^5^ PFU LCMV-Arm or 2 × 10^6^ PFU LCMV-C13. At the indicated time points after infection, blood or spleens were collected for analysis of P14-cell differentiation.

### Flow cytometry

Blood or splenocytes were subjected to red blood cell lysis, washed with FACS buffer, and surface stained with fluorophore-conjugated primary antibodies. All antibodies were purchased from BioLegend: CD90.1 (PerCP/Cy5.5, 202516; FITC, 202503; PE, 205903; BV510, 202535; BV421, 202529), CD27-BV605 (124249), KLRG1 (BV421, 138414; APC, 138412), CD127-PE (135010), and CD8-BV510 (100752). Samples were acquired on a Beckman CytoFLEX S flow cytometer and analyzed with FlowJo software. The sequential gating strategy used to identify P14 donor cells, reporter-positive cells, and phenotypic subsets is shown in **Supplementary Figure 1**.

### Statistical analysis

FACS data were analyzed using Microsoft Excel and GraphPad Prism. Unless otherwise indicated, comparisons between two independent groups were performed using two-tailed unpaired Student’s t-tests. **Figure 1B** was analyzed using ordinary one-way ANOVA followed by Dunnett’s multiple-comparisons test, with the naïve group as the control. **Figure 1C** was analyzed using repeated-measures one-way ANOVA with Geisser–Greenhouse correction followed by Tukey’s multiple-comparisons test. For **Figure 1B**, residual normality and equality of variance were assessed using Prism diagnostics. Data are presented as mean values with individual data points representing biologically independent mice. The statistical test and P value for each comparison are indicated in the corresponding figure or figure legend. P < 0.05 was considered statistically significant.

**Figure 1:**
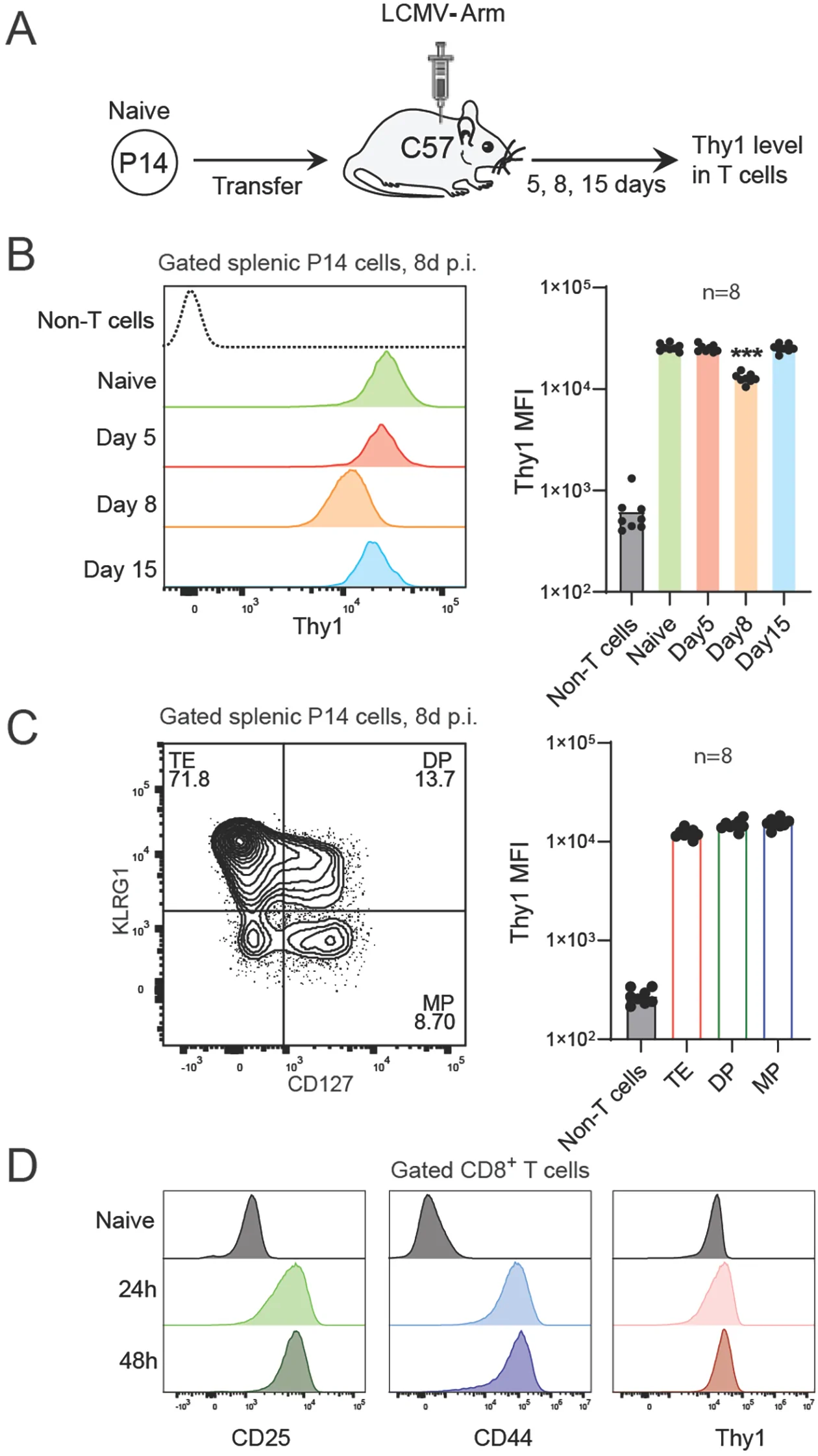
Thy1 expression during CD8^+^ T cell activation and differentiation. **(A)** Experimental design. **(B)** Representative FACS plots and quantification of Thy1 levels in CD8^+^ T cells at different differentiation stages. **(C)** Representative plots and quantification of Thy1 levels in distinct CD8^+^ T cell subsets on day 8 post-infection. **(D)** FACS plots showing CD25, CD44, and Thy1 expression during CD8^+^ T cell activation. Each dot represents one biologically independent mouse and bars show mean values. For B, ordinary one-way ANOVA followed by Dunnett’s multiple-comparisons test, with the naïve group as the control. For C, repeated-measures one-way ANOVA with Geisser–Greenhouse correction followed by Tukey’s multiple-comparisons test. ***P < 0.001.

## Results

### Thy1 expression remains stable during CD8^+^ T cell differentiation

We first monitored Thy1 expression during CD8^+^ T cell differentiation using the P14 model, in which CD8^+^ T cells express a T cell receptor specific for the LCMV GP33 peptide. P14 CD8^+^ T cells were transferred into congenically mismatched host mice, followed by LCMV-Arm infection to initiate differentiation (**Figure 1A**). We analyzed naïve cells, day 5 cells, when KLRG1 expression begins to emerge, day 8 cells at the peak of infection, and day 15 cells during memory formation. Thy1 was highly expressed at all stages relative to non-lymphocytes. Thy1 MFI was similar between naïve cells and cells at days 5 and 15 but was lower at day 8 (**Figure 1B**). At day 8, Thy1 expression remained high across terminal-effector, double-positive, and memory-precursor subsets. A modest subset-associated gradient was detected, with MFI lowest in terminal-effector cells, intermediate in double-positive cells, and highest in memory-precursor cells; however, the absolute difference between double-positive and memory-precursor cells was small (**Figure 1C**). Thus, despite statistically detectable subset-associated variation (Tukey-adjusted P < 0.001), Thy1 expression remained high throughout the differentiation stages and subsets examined. During *in vitro* activation, CD25 and CD44 were strongly upregulated, whereas Thy1 expression remained stable (**Figure 1D**). Together, these data show that Thy1 expression remains high across the CD8^+^ T cell differentiation stages examined.

### Construction of retroviral vectors using Thy1.1 or Thy1.2 reporters

To develop a Thy1 reporter system for the adoptive transfer of mouse CD8^+^ T cells, we focused on retroviral vectors that have successfully been used for the efficient transduction of mouse T cells. As such, the MIGR1 retroviral vector backbone was utilized for the constitutive expression of Thy1.1 or Thy1.2. To assess the usability and flexibility of Thy1 reporters, we created two versions of Thy1-based retroviral vectors. One is an RNAi-oriented Thy1.1 reporter that is independently expressed from a PGK promoter for reporting microRNA-based shRNA expression, and the other is an overexpression-oriented Thy1.2 reporter that is expressed together with the target gene cDNA via a bicistronic IRES element (**Figure 2A**). To observe the RNAi or overexpression effect on CD8^+^ T cell differentiation, we constructed *Tbx21* shRNA or cDNA into these vectors, as T-bet, a transcription factor encoded by the *Tbx21* gene, plays an essential role in CD8^+^ differentiation (13). After transfection of 293T cells for retrovirus production, we easily observed Thy1.1 or Thy1.2 expression in 293T cells (**Figure 2B**). To test these viruses’ function in CD8^+^ T cells, Thy1.2^+/+^ or Thy1.1^+/+^ Naive CD8^+^ T cells were transiently activated and then transduced with the Thy1.1-reporter or Thy1.2-reporter virus, respectively, for 48 hours. Flow cytometry analysis showed that exogenous Thy1.1 or Thy1.2 proteins were readily detected after transduction (**Figures 2C, D**, left). These reporter-positive cells accurately recorded the T-bet loss or gain due to the expression of *Tbx21* shRNA or cDNA, respectively (**Figures 2C, D**, right). To further demonstrate the flexibility of the Thy1 reporter, especially for multi-color FACS, we detected Thy1 reporter-positive cells using Thy1 antibodies conjugated with different fluorophores. As expected, all these antibodies faithfully reported the *Tbx21* RNAi effect (**Figure 2E**). Together, our data indicate that the Thy1 reporters reliably monitor CD8^+^ T cell transgene status.

**Figure 2:**
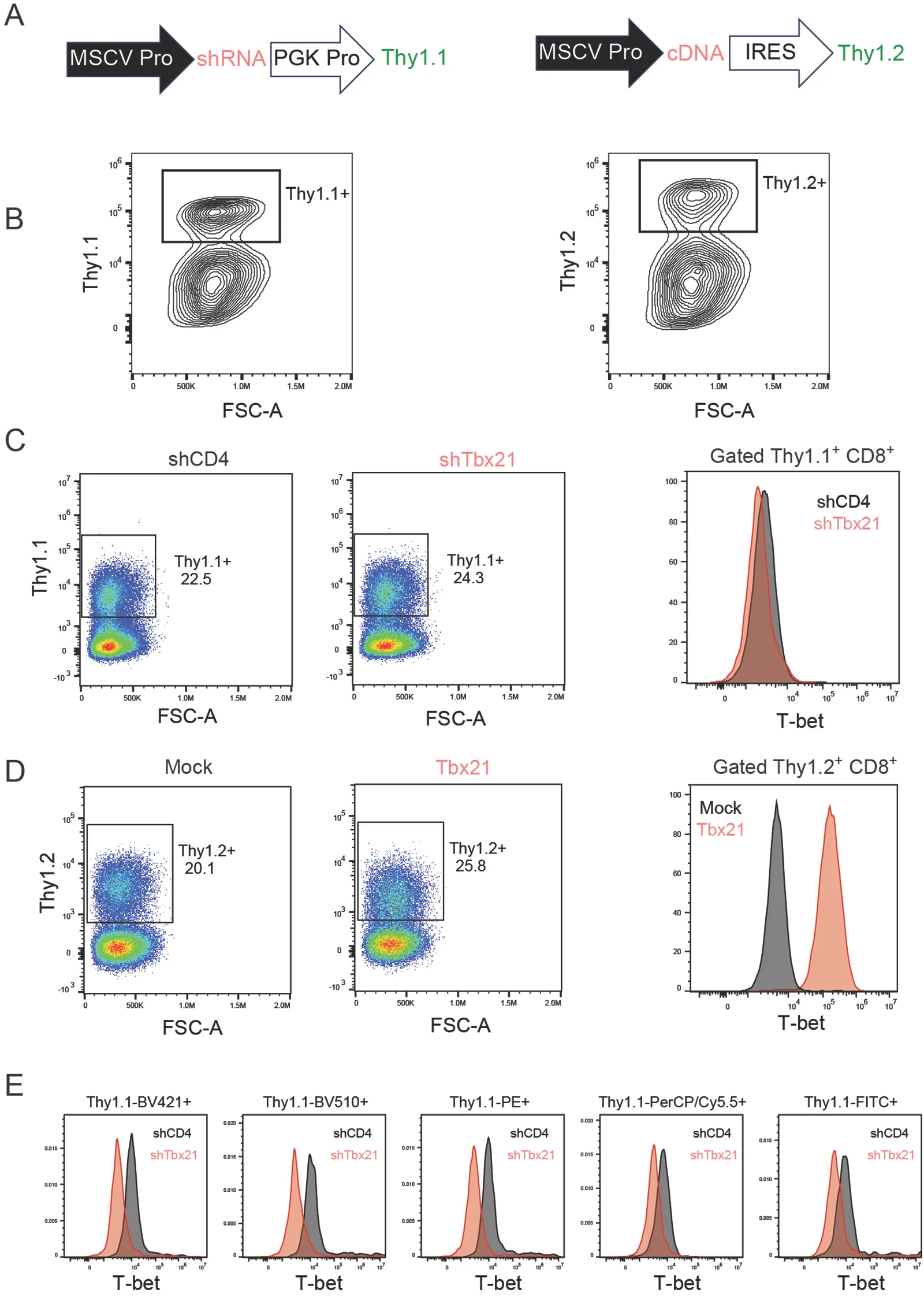
Development and characterization of Thy1 reporter retroviral vectors. **(A)** Retroviral vectors using a Thy1.1 reporter for RNAi or a Thy1.2 reporter for overexpression. **(B)** Representative plots showing Thy1.1 or Thy1.2 reporter expression in 293T cells. **(C)** Representative plots of Thy1.1 reporter expression and the corresponding *Tbx21* RNAi effect. **(D)** Representative plots of Thy1.2 reporter expression and the corresponding *Tbx21* overexpression effect. **(E)** *Tbx21* RNAi detected with multiple fluorophore-conjugated Thy1.1 antibodies.

### Thy1.1 reporter captures phenotypes associated with T-bet loss of function during CD8^+^ T cell differentiation

Next, we tried to apply our Thy1 reporter system for the *in vivo* study of gene function in CD8^+^ T cell differentiation. We first chose a *Tbx21* loss-of-function model to evaluate the Thy1 reporter. To this end, transiently activated P14 CD8^+^ T cells with a Thy1.2 congenic marker, identical to wild-type C57BL/6, were transduced with a retrovirus carrying *Tbx21* shRNA (7, 14) and a Thy1.1 reporter and were adoptively transferred to naive C57BL/6 mice for LCMV-Arm infection, which initiates P14 differentiation (**Figure 3A**). We analyzed P14 cell differentiation status on day 8 post-infection (p.i.). We first examined the P14 cells in the lymphoid organ. As anticipated, the splenic P14 cells expressing CD4 shRNA (the control group) were dominated by TE subsets with a small fraction of DP and MP (**Figure 3B**). In sharp contrast, most *Tbx21*-knockdown P14 cells were CD127^hi^/KLRG1^lo^, a typical characteristic of MP. The frequency of DP subsets, recently described as another type of memory precursor cell (14), was also increased after T-bet loss (**Figure 3B**). Moreover, CD27, a memory precursor-specific but not short-lived effector cell-specific marker, was significantly upregulated by *Tbx21* RNAi (**Figure 3C**). These observations indicate that the loss of T-bet greatly impaired the differentiation of terminal effector cells, with a concomitant increase in memory precursors. We next determined the differentiation of circulating P14 cells. Resembling their splenic counterpart, these cells suffered a loss of TE subsets and favored the differentiation of DP and MP (**Figure 3D**) and CD27^hi^ cells formation (**Figure 3E**). In sum, the Thy1 reporter accurately records transgene function in adoptively transferred CD8^+^ T cells *in vivo*.

**Figure 3:**
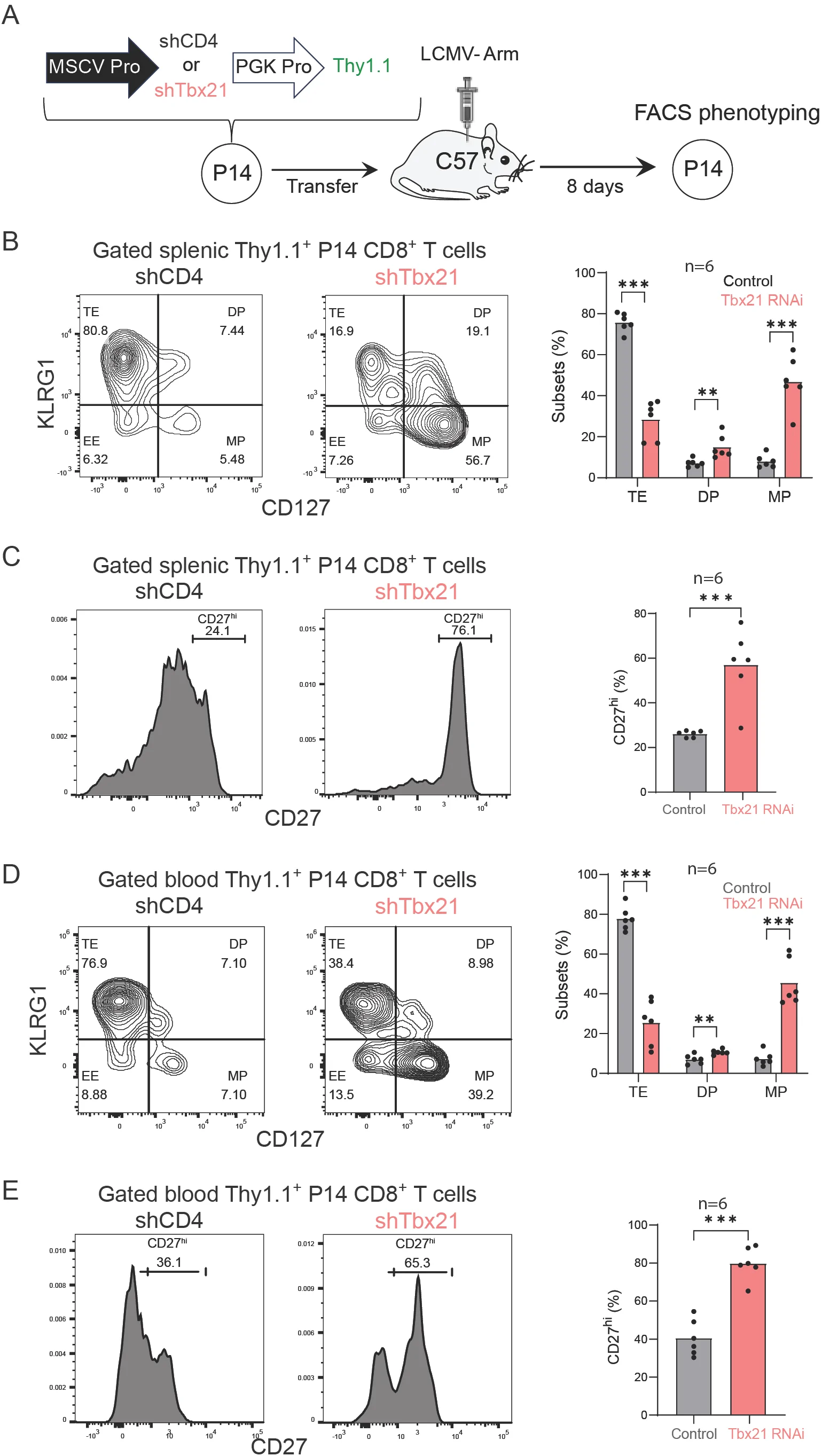
Thy1.1 reporter-based *Tbx21* RNAi during CD8^+^ T cell differentiation *in vivo*. **(A)** Experimental design. **(B, C)** Representative plots and quantification of CD127/KLRG1 **(B)** and CD27 **(C)** staining in splenic Thy1.1 reporter-positive P14 CD8^+^ T cells. **(D, E)** Representative plots and quantification of CD127/KLRG1 **(D)** and CD27 **(E)** staining in circulating Thy1.1 reporter-positive P14 CD8^+^ T cells. Each dot represents one biologically independent mouse and bars show mean values. **(B, D)**, two-tailed unpaired Student’s t-tests with Holm–Šídák correction; **(C, E)**, two-tailed unpaired Student’s t-tests. **P < 0.01 and ***P < 0.001.

### T-bet gain of function in differentiating CD8^+^ T cells is faithfully reported by the Thy1.2 reporter

We next evaluated the Thy1 reporter in a T-bet gain-of-function model. Because GFP is routinely used to label transduced cells in adoptive-transfer experiments, we included it as a reference reporter. Thy1.1^+/+^ P14 cells expressing *Tbx21* cDNA or mock control, together with either the GFP or Thy1.2 reporter, were transferred into naive Thy1.1^+/+^ host mice and analyzed after LCMV-Arm infection (**Figure 4A**). In the Thy1.2 reporter group, approximately 80% of mock-transduced P14 cells were terminal effector cells (**Figure 4B**). *Tbx21* overexpression increased terminal-effector differentiation to more than 95% and reduced the double-positive and memory-precursor populations from 5-10% in mock-transduced cells to less than 0.5%. GFP-reporter cells showed the same *Tbx21*-associated phenotype (**Figure 4C**). These findings show that the Thy1 reporter captures the expected phenotype associated with T-bet overexpression in adoptively transferred CD8^+^ T cells.

**Figure 4:**
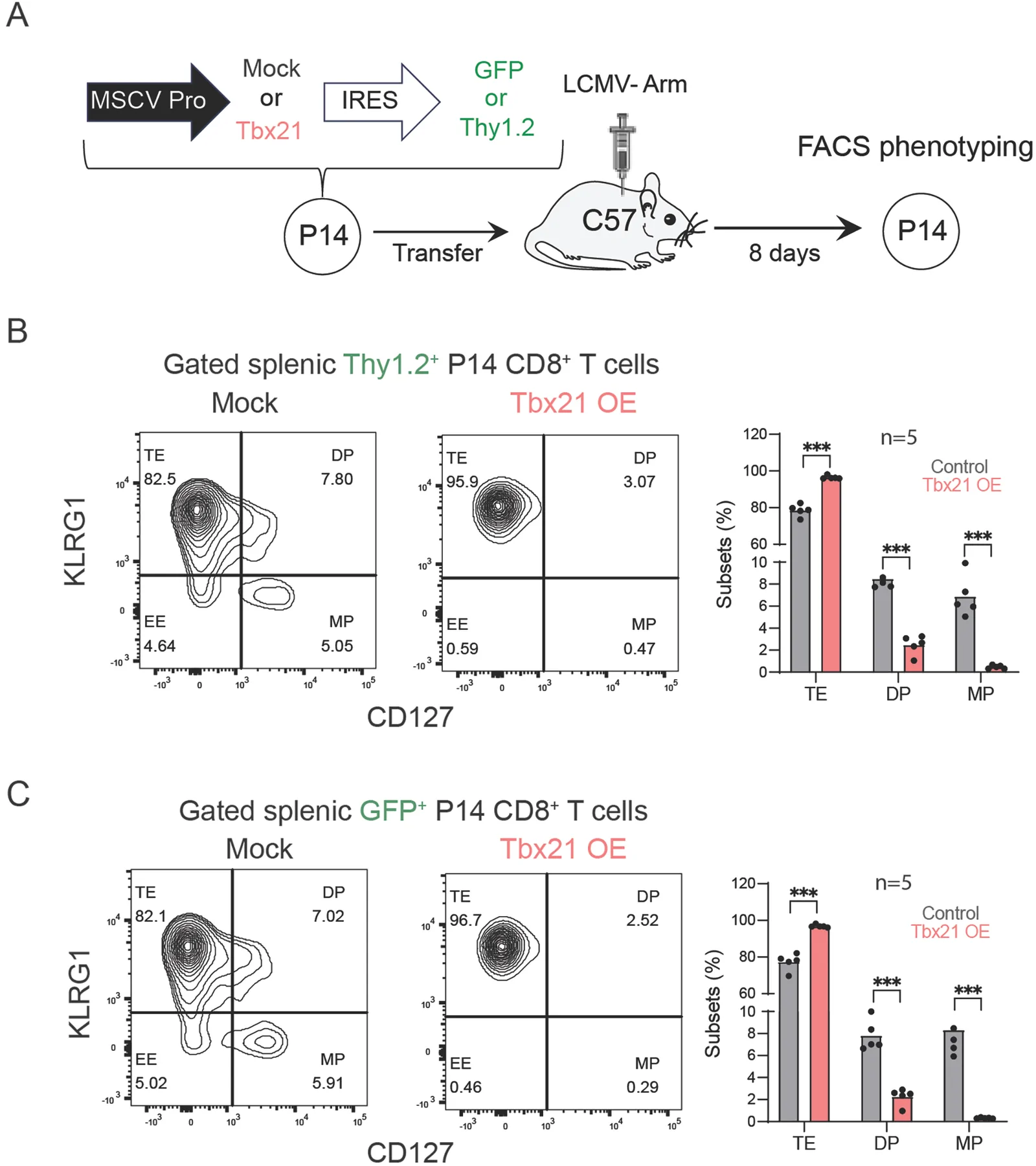
Thy1.2 reporter-based *Tbx21* overexpression during CD8^+^ T cell differentiation *in vivo*. **(A)** Experimental design. **(B, C)** Representative plots and quantification of CD127/KLRG1 staining in splenic P14 CD8^+^ T cells expressing the Thy1.2 **(B)** or GFP **(C)** reporter. Each dot represents one biologically independent mouse and bars show mean values. Unpaired Student’s t-tests with Holm–Šídák correction. ***P < 0.001.

### CD8^+^ T cell differentiation is not measurably altered by forced expression of exogenous Thy1

The key characteristic of a reliable marker to label adoptively transferred T cells is not only its detectability but also its functional neutrality during cell differentiation. Thy1 has been implicated in T cell development in the bone marrow and thymus (15). However, little is known about its role in peripheral T cell differentiation. To investigate if exogenous Thy1 expression might influence CD8^+^ T cell differentiation, we compared the differentiation status of P14 cells expressing empty vectors with GFP or Thy1.2 as the reporter. The three key differentiation subsets of differentiating P14 cells—TE, DP, and MP—demonstrated similar frequencies irrespective of their reporter type (**Figure 5A**), indicating that CD8^+^ cell subset differentiation was not measurably altered by forced Thy1 expression. We also quantified P14-cell numbers in GFP-mock and Thy1-mock groups and did not observe a statistically significant difference between the groups (**Figure 5B**), supporting the absence of a detectable effect of Thy1 on the measured CD8^+^ differentiation outcomes. Finally, we assessed Thy1 reporter expression in CD8^+^ T cells. Under matched staining and acquisition conditions, endogenous Thy1 exhibited an approximately tenfold higher MFI than exogenous Thy1 in the same reporter-positive cells (**Figures 5C, D**), indicating that exogenous reporter expression contributes only a minor component of the total Thy1 signal in this experimental setting.

**Figure 5:**
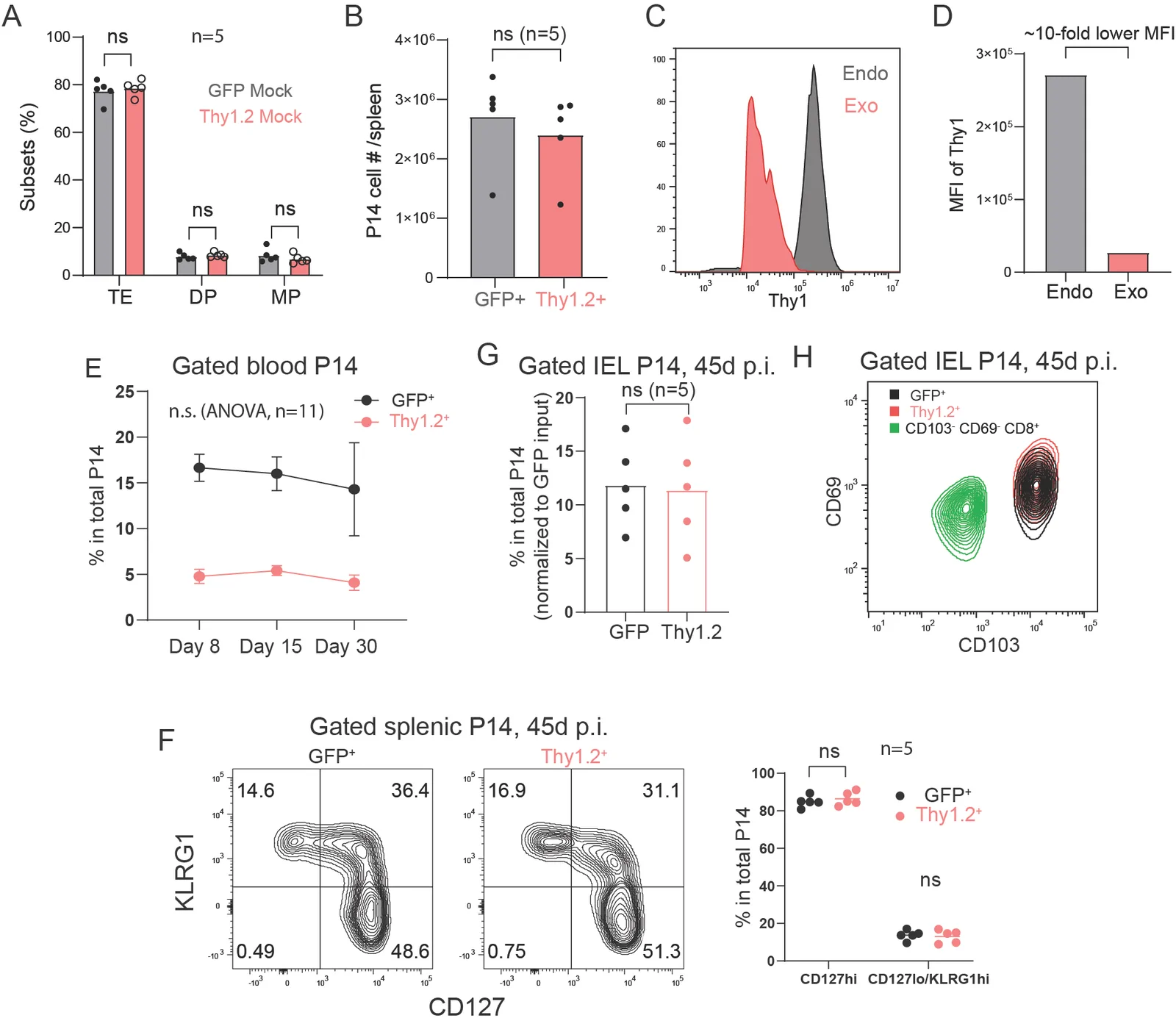
Thy1 reporter is functionally neutral and remains detectable during acute infection and memory. **(A)** Comparison of differentiation-subset frequencies in mock-transduced P14 CD8^+^ T cells expressing the Thy1.2 or GFP reporter, using data from **Figures 4B, C**, respectively. **(B)** Total splenic P14-cell numbers in mice receiving mock-transduced cells expressing the Thy1.2 or GFP reporter. **(C, D)** Representative FACS histograms **(C)** and corresponding mean fluorescence intensity (MFI) values **(D)** for endogenous and exogenous Thy1 in transduced CD8^+^ T cells. **(E)** Longitudinal analysis of blood reporter-positive P14-cell frequencies. **(F)** Memory-phenotype frequencies among reporter-positive P14 cells in the spleen at day 45 post-infection. **(G, H)** Frequency and CD103/CD69 phenotype of reporter-positive P14 cells among intraepithelial lymphocytes at day 45 post-infection. Each dot represents one biologically independent mouse and bars show mean values. **(E)**, two-way repeated-measures ANOVA; **(A, F)**, two-tailed unpaired Student’s t-tests with Holm–Šídák correction; **(B, G)**, two-tailed unpaired Student’s t-tests. ns, not significant.

To further assess Thy1 reporter stability, we performed a longitudinal analysis of the differentiation and persistence of Thy1.2 reporter-positive P14 cells, using GFP-positive P14 cells as a reference. The frequency of circulating Thy1.2 reporter-positive P14 cells over the course of differentiation was comparable to that of GFP-positive cells (**Figure 5E**). At day 45 post-infection, Thy1.2 reporter-positive and GFP-positive cells showed similar frequencies of CD127^hi^ and CD127lo/KLRG1^hi^ populations (**Figure 5F**). We also evaluated intraepithelial lymphocyte P14 cells. Thy1.2 reporter-positive and GFP-positive cells were recovered at comparable frequencies (**Figure 5G**), and both populations were predominantly CD103^+^CD69^+^, consistent with a tissue-resident memory phenotype (**Figure 5H**). These findings support stability of the Thy1.2 reporter during the acute response and into the memory phase.

### Thy1 reporter captures CD8^+^ T cell differentiation during chronic infection

Because the preceding studies used an acute infection model, we next assessed whether the Thy1 reporter could also report CD8^+^ T cell differentiation during chronic infection. P14 cells expressing the Thy1.2 or GFP reporter were analyzed 10 days after LCMV-C13 infection. PD-1, TIM-3, CD101, and CX3CR1 were induced during chronic infection. PD-1 MFI and the frequencies of TIM-3^hi^, CD101^hi,^ and CX3CR1^hi^ cells were comparable between Thy1.2-positive and GFP-positive cells (**Figure 6A**). We then assessed the effect of *Tbx21* overexpression in this setting. *Tbx21*-overexpressing cells showed exhaustion-associated marker profiles comparable to those of mock-transduced cells (**Figure 6B**) but increased KLRG1 expression, consistent with the established role of T-bet in promoting KLRG1 expression (**Figure 6C**). These data further support the use of Thy1 as a reporter of CD8^+^ T cell differentiation in this chronic-infection model.

**Figure 6:**
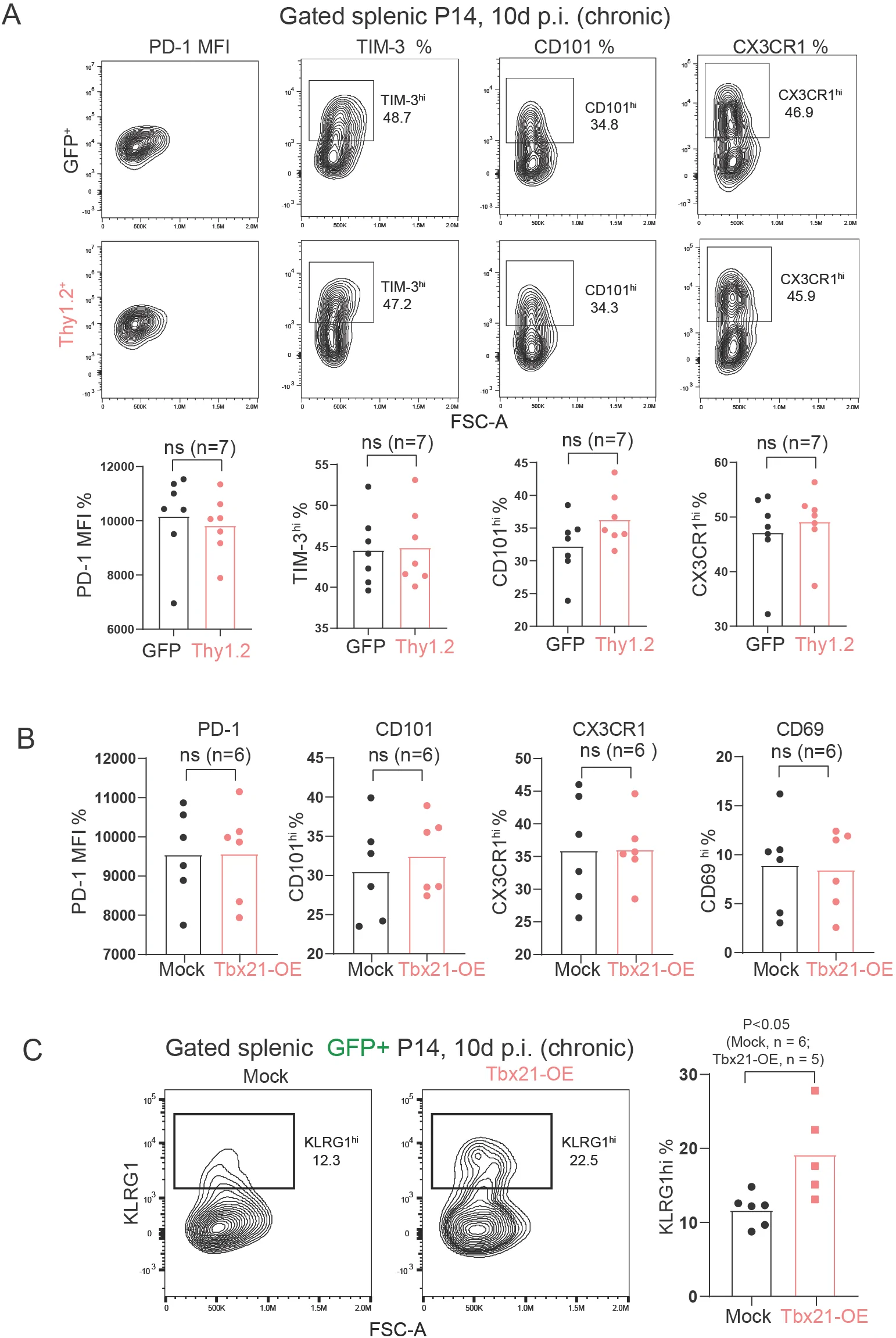
Thy1 reporter captures CD8^+^ T cell differentiation during chronic LCMV infection. **(A)** Representative FACS plots and quantification of exhaustion-associated markers among splenic GFP-positive and Thy1.2-positive P14 cells 10 days after LCMV-C13 infection. **(B)** Quantification of exhaustion-associated markers in mock-transduced and *Tbx21*-overexpressing P14 cells. **(C)** Representative plots and quantification of KLRG1^hi^ cells among GFP-positive mock-transduced and *Tbx21*-overexpressing P14 cells. Each dot represents one biologically independent mouse and bars show mean values. Quantitative comparisons were analyzed using two-tailed unpaired Student’s t-tests. Sample sizes and P values are indicated in the corresponding panels; *P < 0.05 as indicated. ns, not significant.

## Discussion

The data presented here establish a Thy1-based retroviral reporter system for congenic background-independent adoptive transfer and CD8^+^ T cell differentiation studies. By decoupling donor-host discrimination from endogenous congenic backgrounds, the system can reduce the breeding burden associated with establishing congenically mismatched donor or recipient mice.

An ideal reporter for adoptive T cell transfer should be readily detectable and compatible with live-cell magnetic enrichment. Although fluorescent proteins such as GFP are widely used for tracking transduced cells, they exhibit immunogenicity in certain immunocompetent mouse strains or experimental settings (16, 17), which can confound data on T cell survival and differentiation (18, 19). Moreover, because fluorescent proteins are intracellular, they cannot be targeted by antibodies on live cells, thereby precluding magnetic bead-based enrichment of donor populations. Thy1 therefore provides a surface-accessible alternative when live-cell antibody-based enrichment is required in adoptive-transfer studies.

Although Thy1 has been sporadically used as a retroviral reporter in previous adoptive transfer and immunotherapy studies (9, 10, 12), these applications primarily used Thy1 to identify, enrich, or track genetically modified T cells rather than to evaluate its effect on CD8^+^ T-cell differentiation. Because endogenous Thy1 participates in T-cell signaling and development, forced exogenous expression could conceivably influence differentiation programs (20, 21). In the present study, exogenous Thy1.1 or Thy1.2 expression did not measurably alter lineage-defining marker expression, terminal-effector, double-positive, or memory-precursor subset distribution, cellular expansion, or memory formation. These findings support the functional neutrality of the reporter in the CD8^+^ T-cell settings examined.

Furthermore, under matched staining and acquisition conditions, exogenous Thy1 yielded an MFI approximately tenfold lower than endogenous Thy1. This relative MFI comparison indicates that exogenous Thy1 contributed a small component of the measured total Thy1 signal in the same reporter-positive cells. Consistent with the high endogenous expression of Thy1 on T cells (21), this relative MFI difference may help explain why no effect of forced Thy1 expression was detected for the assessed CD8^+^ T cell differentiation. The remarkable stability of Thy1 expression from the naive stage through effector and memory phases further supports its suitability for long-term studies.

The utility of this platform was confirmed through both loss-of-function (shRNA) and gain-of-function (cDNA) modulation of the transcription factor T-bet. In the well-established P14 TCR-transgenic LCMV model, Thy1 reporters faithfully recapitulated expected T-bet-dependent phenotypes (22, 23) in both splenic and circulating CD8^+^ T cells. These results support the use of the system for genetic interrogation of CD8^+^ T cell fate decisions.

CD45, another widely used congenic marker that shares several advantageous features with Thy1—including surface expression and the broad availability of multiple fluorophore-conjugated antibodies against CD45.1 and CD45.2 isoforms—was not selected for this reporter system. We chose Thy1 over CD45 primarily due to practical limitations in retroviral packaging. The CD45 cDNA is substantially longer than Thy1 cDNA (24) and its incorporation into a retroviral backbone would exceed optimal proviral size limits, resulting in markedly reduced viral titers and transduction efficiency. Similarly, although lentiviral vectors are widely used in other applications, we opted for retroviral backbones because lentiviruses exhibit limited efficiency in transducing and programming specific subsets of primary mouse T cells in this experimental context (25, 26).

These findings should be interpreted within the scope of the P14 CD8^+^ T-cell model tested during acute LCMV-Arm and chronic LCMV-C13 infection. Because the P14-LCMV system is widely used to study antigen-specific CD8^+^ T-cell differentiation, the present findings may be informative for other antigen-specific CD8^+^ T-cell systems. Further evaluation in additional models would define the broader applicability of the platform. Its stability and functional neutrality under prolonged or repeated antigen exposure, in tumor microenvironments or other tissue and disease settings, and in other T-cell subsets and human T cells remain to be determined. In addition, potential isoform-specific immune effects of the reporter were not directly evaluated in the present study.

## Conclusion

In summary, the Thy1 retroviral reporter platform enables adoptive-transfer studies without pre-establishing a congenic mismatch between donor and recipient mice. In the acute and chronic P14-LCMV models examined here, Thy1 reporter expression did not measurably alter the assessed differentiation phenotypes and provided a stable surface marker for tracking transduced cells. Thy1.1 may be preferable when stronger staining with available antibodies is required. The platform provides a practical tool for genetic studies of CD8^+^ T cell differentiation in mouse models.

## Data Availability

The original contributions presented in the study are included in the article/**Supplementary Material**, further inquiries can be directed to the corresponding author/s.

## Supplementary material

Supplementary Figure 1 Flow-cytometry gating strategy and retroviral transduction efficiency. Representative sequential gating strategy used to identify P14 donor cells, reporter-positive cells, and the indicated phenotypic subsets. Retroviral transduction efficiencies for the indicated Thy1 reporter constructs are shown.

## References

[1] Zander R Kasmani MY Chen Y Topchyan P Shen J Zheng S . Tfh-cell-derived interleukin 21 sustains effector CD8(+) T cell responses during chronic viral infection. Immunity. (2022) 55:475–93:e5. doi: 10.1016/j.immuni.2022.01.018 35216666 PMC8916994

[2] Redeker A Arens R . Improving adoptive T cell therapy: The particular role of T cell costimulation, cytokines, and post-transfer vaccination. Front Immunol. (2016) 7:345. doi: 10.3389/fimmu.2016.00345 27656185 PMC5011476

[3] Edwards-Hicks J Apostolova P Buescher JM Maib H Stanczak MA Corrado M . Phosphoinositide acyl chain saturation drives CD8(+) effector T cell signaling and function. Nat Immunol. (2023) 24:516–30. doi: 10.1038/s41590-023-01419-y 36732424 PMC10908374

[4] Restifo NP Dudley ME Rosenberg SA . Adoptive immunotherapy for cancer: Harnessing the T cell response. Nat Rev Immunol. (2012) 12:269–81. doi: 10.1038/nri3191 22437939 PMC6292222

[5] Naviaux RK Costanzi E Haas M Verma IM . The pCL vector system: Rapid production of helper-free, high-titer, recombinant retroviruses. J Virol. (1996) 70:5701–5. doi: 10.1128/JVI.70.8.5701-5705.1996 8764092 PMC190538

[6] Buchholz VR Flossdorf M Hensel I Kretschmer L Weissbrich B Graf P . Disparate individual fates compose robust CD8+ T cell immunity. Science. (2013) 340:630–5. doi: 10.1126/science.1235454 23493420

[7] Wang D Xiu J Zhao J Luo J . miR-AB, a miRNA-based shRNA viral toolkit for multicolor-barcoded multiplex RNAi at a single-cell level. EMBO Rep. (2022) 23:e53691. doi: 10.15252/embr.202153691 35201651 PMC8982575

[8] Moon JJ Chu HH Hataye J Pagan AJ Pepper M McLachlan JB . Tracking epitope-specific T cells. Nat Protoc. (2009) 4:565–81. doi: 10.1038/nprot.2009.9 19373228 PMC3517879

[9] Kadyrzhanova G Tamai M Sarkar S Kalra RS Ishikawa H . Aging impairs CD8 T cell responses in adoptive T-cell therapy against solid tumors. Front Immunol. (2025) 16:1484303. doi: 10.3389/fimmu.2025.1484303 39925817 PMC11803149

[10] Rabinovich BA Ye Y Etto T Chen JQ Levitsky HI Overwijk WW . Visualizing fewer than 10 mouse T cells with an enhanced firefly luciferase in immunocompetent mouse models of cancer. PNAS. (2008) 105:14342–6. doi: 10.1073/pnas.0804105105 18794521 PMC2567214

[11] Kerkar SP Sanchez-Perez L Yang S Borman ZA Muranski P Ji Y . Genetic engineering of murine CD8+ and CD4+ T cells for preclinical adoptive immunotherapy studies. J Immunotherapy. (2011) 34:343–52. doi: 10.1097/CJI.0b013e3182187600 21499127 PMC3100770

[12] Gautam S Fioravanti J Zhu W Le Gall JB Brohawn P Lacey NE . The transcription factor c-Myb regulates CD8(+) T cell stemness and antitumor immunity. Nat Immunol. (2019) 20:337–49. doi: 10.1038/s41590-018-0311-z 30778251 PMC6489499

[13] Sullivan BM Juedes A Szabo SJ von Herrath M Glimcher LH . Antigen-driven effector CD8 T cell function regulated by T-bet. PNAS. (2003) 100:15818–23. doi: 10.1073/pnas.2636938100 14673093 PMC307651

[14] Herndler-Brandstetter D Ishigame H Shinnakasu R Plajer V Stecher C Zhao J . KLRG1(+) effector CD8(+) T cells lose KLRG1, differentiate into all memory T cell lineages, and convey enhanced protective immunity. Immunity. (2018) 48:716–29:e8. doi: 10.1016/j.immuni.2018.03.015 29625895 PMC6465538

[15] Killeen N . T-cell regulation: Thy-1 - hiding in full view. Curr Biol CB. (1997) 7:R774–7. doi: 10.1016/s0960-9822(06)00402-7 9382830

[16] Manjunath N Shankar P Stockton B Dubey PD Lieberman J von Andrian UH . A transgenic mouse model to analyze CD8(+) effector T cell differentiation *in vivo*. PNAS. (1999) 96:13932–7. doi: 10.1073/pnas.96.24.13932 10570176 PMC24168

[17] Gambotto A Dworacki G Cicinnati V Kenniston T Steitz J Tuting T . Immunogenicity of enhanced green fluorescent protein (EGFP) in BALB/c mice: Identification of an H2-Kd-restricted CTL epitope. Gene Ther. (2000) 7:2036–40. doi: 10.1038/sj.gt.3301335 11175316

[18] Stripecke R Carmen Villacres M Skelton D Satake N Halene S Kohn D . Immune response to green fluorescent protein: Implications for gene therapy. Gene Ther. (1999) 6:1305–12. doi: 10.1038/sj.gt.3300951 10455440

[19] Yang C Hao F He J Lu T Klein RL Zhao LR . Sequential adeno-associated viral vector serotype 9-green fluorescent protein gene transfer causes massive inflammation and intense immune response in rat striatum. Hum Gene Ther. (2016) 27:528–43. doi: 10.1089/hum.2015.083 26847924

[20] Leyton L Schneider P Labra CV Ruegg C Hetz CA Quest AF . Thy-1 binds to integrin beta(3) on astrocytes and triggers formation of focal contact sites. Curr Biol CB. (2001) 11:1028–38. doi: 10.1016/s0960-9822(01)00262-7 11470407

[21] Haeryfar SM Hoskin DW . Thy-1: More than a mouse pan-T cell marker. J Immunol. (2004) 173:3581–8. doi: 10.4049/jimmunol.173.6.3581 15356100

[22] Intlekofer AM Takemoto N Kao C Banerjee A Schambach F Northrop JK . Requirement for T-bet in the aberrant differentiation of unhelped memory CD8+ T cells. J Exp Med. (2007) 204:2015–21. doi: 10.1084/jem.20070841 17698591 PMC2118697

[23] Xin A Masson F Liao Y Preston S Guan T Gloury R . A molecular threshold for effector CD8(+) T cell differentiation controlled by transcription factors Blimp-1 and T-bet. Nat Immunol. (2016) 17:422–32. doi: 10.1038/ni.3410 26950239 PMC5779087

[24] Shen FW Saga Y Litman G Freeman G Tung JS Cantor H . Cloning of Ly-5 cDNA. PNAS. (1985) 82:7360–3. doi: 10.1073/pnas.82.21.7360 3864163 PMC391344

[25] Baumann JG Unutmaz D Miller MD Breun SK Grill SM Mirro J . Murine T cells potently restrict human immunodeficiency virus infection. J Virol. (2004) 78:12537–47. doi: 10.1128/JVI.78.22.12537-12547.2004 15507641 PMC525105

[26] Tsurutani N Yasuda J Yamamoto N Choi BI Kadoki M Iwakura Y . Nuclear import of the preintegration complex is blocked upon infection by human immunodeficiency virus type 1 in mouse cells. J Virol. (2007) 81:677–88. doi: 10.1128/JVI.00870-06 17079325 PMC1797461

